# Zinc Acetate Dihydrate Tablet-Associated Gastritis Occurring in a Post-Hematopoietic Stem Cell Transplant Recipient

**DOI:** 10.1155/2022/4637707

**Published:** 2022-12-31

**Authors:** Masaya Iwamuro, Takehiro Tanaka, Akifumi Matsumura, Seiji Kawano, Yoshiro Kawahara, Hiroyuki Okada

**Affiliations:** ^1^Department of Gastroenterology and Hepatology, Okayama University Graduate School of Medicine, Dentistry and Pharmaceutical Sciences, Okayama 700-8558, Japan; ^2^Department of Pathology, Okayama University Graduate School of Medicine, Dentistry and Pharmaceutical Sciences, Okayama 700-8558, Japan; ^3^Department of Hematology and Oncology, Okayama University Graduate School of Medicine, Dentistry and Pharmaceutical Sciences, Okayama 700-8558, Japan; ^4^Department of Practical Gastrointestinal Endoscopy, Okayama University Hospital, Okayama 700-8558, Japan

## Abstract

A 65-year-old Japanese woman underwent umbilical cord blood transplantation for acute myeloid leukemia. Zinc acetate dihydrate tablets were administered for hypozincemia after transplantation, and vomiting and appetite loss occurred soon thereafter. Esophagogastroduodenoscopy revealed mucosal redness, erosion, white coat adhesion, and ulcers. Although graft-versus-host disease, intestinal transplant-associated microangiopathy, and cytomegalovirus infection were considered as possible causes, we diagnosed the patient with zinc acetate dihydrate tablet-associated gastric mucosal alterations based on the endoscopic features. This case reinforces the notion that medication-associated gastric lesions should be suspected in patients taking zinc acetate dihydrate tablets.

## 1. Introduction

Zinc is one of the most important trace elements in the body. A shortage of zinc may result from insufficient zinc intake, malabsorption, increased zinc demand, and increased excretion. Low-birth-weight infants, pregnant women, and the elderly are prone to zinc deficiency. Chronic liver disease, short bowel syndrome, diabetes, chronic renal disease, long-term use of chelating agents, parenteral nutrition, and tube feeding with insufficient zinc supplementation may also cause zinc deficiency [1–6]. Zinc deficiency leads to taste disorders, dermatitis, hair loss, anemia, stomatitis, male sexual dysfunction, increased susceptibility to infections, and osteoporosis. Zinc acetate dihydrate is prescribed as a supplement for patients with zinc deficiency.

Zinc acetate dihydrate is generally safe and well tolerated. Meanwhile, erosions and ulcers have recently been reported in the stomach of patients taking zinc acetate dihydrate [7, 8]. Herein, we describe a case of a patient who developed gastric ulcers after hematopoietic stem cell transplantation. Although various diseases, such as graft-versus-host disease, transplant-associated microangiopathy, and cytomegalovirus gastritis, had been considered as possible causes, the patient was finally diagnosed with zinc acetate dihydrate tablet-induced gastric mucosal damage.

## 2. Case Report

A 65-year-old Japanese woman was diagnosed with *FMS-like tyrosine kinase 3*-internal tandem duplication mutation-positive acute myeloid leukemia. Treatment with idarubicin and cytarabine was initiated but was ineffective. The patient was treated with gilteritinib. Because her acute myeloid leukemia did not respond to gilteritinib, she underwent umbilical cord blood transplantation after one cycle of treatment with gilteritinib, 5-azacytidine, and venetoclax. The patient complained of dysgeusia after transplantation and blood tests revealed decreased serum zinc levels (66 *μ*g/dL; reference range, 80–130 *μ*g/dL), which was considered to be a possible cause of dysgeusia. Thus, zinc acetate dihydrate tablets were administered for hypozincemia 35 days after transplantation. Vomiting and appetite loss occurred on the day of zinc acetate dihydrate tablet administration. The patient was referred to the department of gastroenterology for investigation of gastrointestinal symptoms. She had been taking amlodipine, lansoprazole, ursodeoxycholic acid, ambroxol, brotizolam, acyclovir, letermovir, *Clostridium butyricum* preparation, and mycophenolate mofetil, in addition to zinc acetate dihydrate tablets.

**Figure 1:**
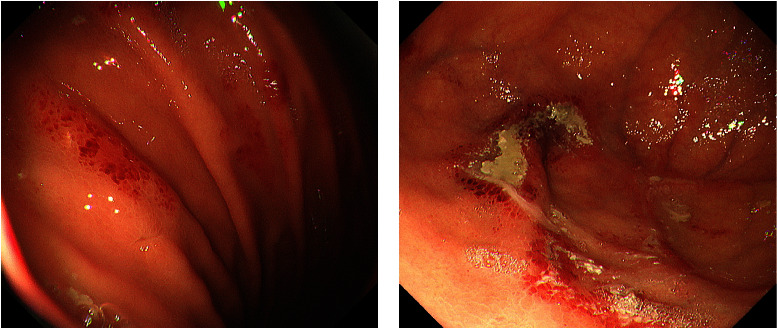
Endoscopic images. (a) Esophagogastroduodenoscopy reveals multiple reddish spots in the gastric body. (b) Shallow ulcers with white coat adhesion in the gastric fornix.

**Figure 2:**
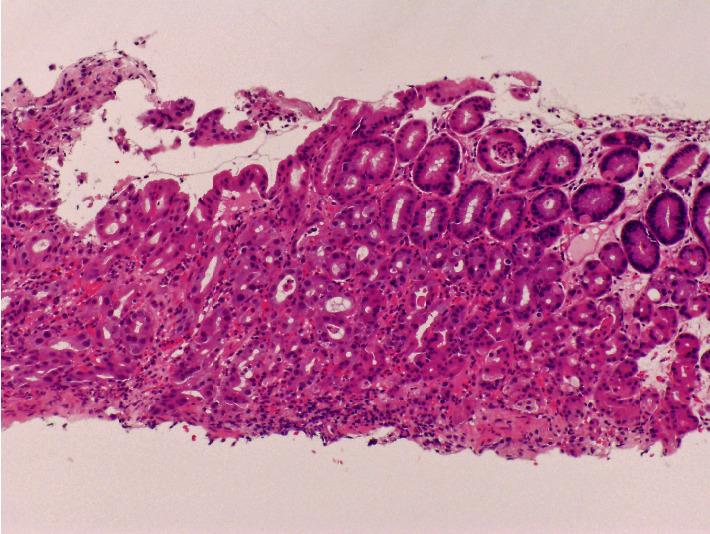
A pathology image. A biopsy from the gastric ulcer shows focal inflammation, erosion, and degenerative epithelium. These features are consistent with pathological features specific to zinc acetate dihydrate tablet-associated gastric lesions.

**Figure 3:**
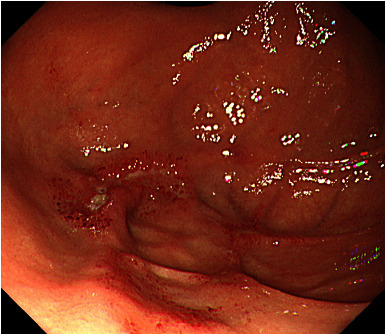
An endoscopy image performed 6 days after cessation of zinc acetate dihydrate tablet intake. Esophagogastroduodenoscopy shows improvement in gastric lesions.

Esophagogastroduodenoscopy performed 41 days after transplantation revealed multiple reddish spots in the gastric body (Figure 1(a)) and shallow ulcers with white coat adhesion in the gastric fornix (Figure 1(b)). Zinc acetate dihydrate tablets were discontinued because we suspected that they were associated with the gastric lesions. As the patient was a hematopoietic stem cell transplant recipient under immunosuppressive treatment, involvement of graft-versus-host disease, intestinal transplant-associated microangiopathy, and cytomegalovirus infection were also considered as differential diagnoses of gastric mucosal damage. However, these findings were ruled out by pathological analysis. A biopsy of one ulcer showed focal inflammation, erosion, and degenerative epithelium (Figure 2), which were consistent with the pathological features specific to zinc acetate dihydrate tablet-associated gastric lesions. Esophagogastroduodenoscopy performed 6 days after cessation of zinc acetate dihydrate tablet intake showed improvement in gastric lesions (Figure 3).

## 3. Discussion

Medications containing the active ingredient zinc commercially available in Japan include polaprezinc and zinc acetate [9]. Polaprezinc has been used for the treatment of gastric ulcers, whereas zinc acetate is prescribed to patients with Wilson's disease or hypozincemia [10]. Although dietary zinc intake is recommended as an initial treatment for hypozincemia, zinc acetate dihydrate is used when dietary therapy is insufficient. In patients with Wilson's disease, chelating agents excrete copper in the body into the urine, whereas zinc acetate dihydrate inhibits the absorption of copper from food and excretes copper into feces. Ingested zinc induces the production of metallothionein in intestinal mucosal epithelial cells, which is the major endogenous copper-binding protein [11]. Patients with Wilson's disease need to avoid copper-rich foods, such as organ meat and shellfish. However, dietary restrictions are usually insufficient to control Wilson's disease on their own. Thus, zinc acetate dihydrate is prescribed in combination with chelating drugs, such as penicillamine and trientine, which have different mechanisms of action, aiming to relax strict dietary restrictions. Zinc acetate dihydrate may also be used as a prophylactic agent in asymptomatic patients with Wilson's disease [12, 13].

Adverse events associated with zinc acetate dihydrate include copper deficiency and increased levels of blood amylase, lipase, and alkaline phosphatase. Gastrointestinal symptoms, such as epigastric discomfort, nausea, vomiting, abdominal pain, diarrhea, and constipation, may occur [14]. In addition, gastric mucosal injury has recently been reported to be associated with zinc acetate dihydrate tablet intake. Kitagawa et al. reported that in three cases of Wilson's disease treated with zinc acetate, lustrous white erosions surrounded by erythematous mucosa were observed in the greater curvature of the gastric corpus [8]. We retrospectively analyzed 47 patients who underwent upper gastrointestinal endoscopy while taking oral zinc acetate dihydrate [7]. Gastric mucosal injury was identified in 29 patients (61.7%), including mucosal redness (93.1%), erosion (90.0%), white coat adhesion (86.2%), and ulcers (31.0%). The most frequently involved area was the middle third of the stomach (96.6%), followed by the upper third (65.5%) and lower third (46.4%). Therefore, ulcers and erosions with white coat and redness of the surrounding mucosa, which were observed in the present case, are typical endoscopic features of zinc acetate dihydrate-associated gastric lesions.

The mechanism of zinc acetate dihydrate-associated gastric lesions has not yet been elucidated. Kitagawa et al. reported that orcein-positive granules were observed in the fundic glands and interstitium of the stomach in three cases of Wilson's disease, suggesting cytotoxicity induced by copper deposition [8]. Although we performed energy-dispersive X-ray analysis of the biopsy specimen from another patient with hypozincemia, no copper or zinc was detected [15]. Possible factors other than copper deposition include the corrosive action of high concentrations of zinc. For instance, oral intake of iron causes bleeding, erosion, and necrosis when in contact with the gastrointestinal mucosa for a long period [16]. Polaprezinc, a complex of zinc and L-carnosine, contains 17 mg of zinc per tablet, giving a daily zinc intake of 34 mg. Regarding zinc acetate dihydrate, 50–150 and 150–250 mg of zinc are administered per day for hypozincemia and Wilson's disease, respectively. Therefore, higher concentrations of zinc, compared with polaprezinc, may damage the gastric mucosa.

Generally, hematopoietic stem cell transplant recipients may develop gastrointestinal lesions due to various causes, such as graft-versus-host disease, transplant-associated microangiopathy, and cytomegalovirus infection [17]. Because each disease requires different treatments, prompt diagnosis of the underlying cause is essential. Although biopsy and pathological analysis are inevitable, this case reinforces that the recognition of representative endoscopic features of zinc acetate dihydrate tablet-associated gastric mucosal alterations enabling appropriate diagnosis and management without delay, particularly when various potential causes can be considered.

## 4. Conclusions

We experienced a case of zinc acetate dihydrate tablet-associated gastric lesions, which presented with typical features of mucosal redness, erosion, white coat adhesion, and ulcers. Medication-associated gastric lesions should be suspected in patients taking zinc acetate dihydrate tablets.

## Data Availability

The data used to support the findings of this study are available from the corresponding author upon reasonable request.
